# Enchondroma Protuberans of Ulnar Bone: A Case Report and Review of Literature

**DOI:** 10.1155/2012/278920

**Published:** 2012-09-06

**Authors:** Afshin Mohammadi, Abbas Hedayati Asl, Mohammad Ghasemi-Rad, Farahnaz Noroozinia

**Affiliations:** ^1^Department of Radiology, Urmia University of Medical Sciences, Urmia, West-Azerbaijan, Iran; ^2^Omid Oncology Center, Urmia University of Medical Sciences, Urmia, Iran; ^3^Department of Pathology, Urmia University of Medical Sciences, Urmia, West-Azerbaijan, Iran

## Abstract

*Introduction*. Enchondroma protuberans is an extremely rare benign cartilaginous bone tumor. We report the first case report of enchondroma protuberans in the forearm. *Presentation of Case*. We report a case of enchondroma protuberans originating in the left ulnar bone of a young woman. A 20-year-old female referred to our hospital complaining of progressive sustained left forearm pain with a radiation to fourth and fifth finger. Conventional radiography revealed a well-defined eccentric osteolytic lesion in the distal diaphysis of ulna with expansion of overlying cortex (without calcification). Magnetic resonance imaging showed a well-defined ovoid intramedullary lesion, which was exophytically protruding from medial surface of left ulnar bone. Histopathology confirmed the diagnosis. *Discussion*. Enchondroma protuberans typically present as a well-defined intramedullary osteolytic lesion that may be accompanied by a fine matricidal calcification. The connection between the intramedullary portion and the exophytic protrusion can be seen well by magnetic resonance imaging. *Conclusion*. Enchondroma protuberans should be considered in the differential diagnosis of osteochondroma, enchondroma, and periosteal chondroid tumors.

## 1. Introduction

Enchondroma protuberans is a rare benign cartilaginous bone tumor. It arises from the intramedullary cavity of long bones, which usually protrudes beyond the cortex. Based on our extensive medical database search, there are only handfuls of cases reported previously [1–10]. Most of the previous reports are in the phalanges or metacarpal bones (Although they supposedly should present in the ribs and humerus) [1–10]. We report a case of enchondroma protuberans originating in the left ulnar bone of a young woman. This is the first case report of enchondroma protuberans in the forearm region.

## 2. Presentation of Case

We present a 20-year-old female who referred to our hospital complaining of progressive sustained left forearm pain with radiation to fourth and fifth finger of one-year duration. There was also a bulging mass, progressively growing on the lower dorsal surface of her left forearm. On physical examination, there was a 1.5 by 1.5 cm firm tender mass on the lower dorsal surface of her left forearm. The skin overlying the lesion was normal and the elbow joint had a normal range of motion. She had no weakness in her hands. Strength was measured 5/5 at wrist and forearm bilaterally. Sensation was normal to light touch, temprature, and crude touch in all five finger. There was no paresthesia, numbness, or tingling. She was able to determine static two-point discrimination of 1 cm in the radial, median, and ulnar nerve distributions of her both hands. All other local physical examinations were normal.

All laboratory studies including blood cell and erythrocyte sedimentation rate were within normal limit.

**Figure 1:**
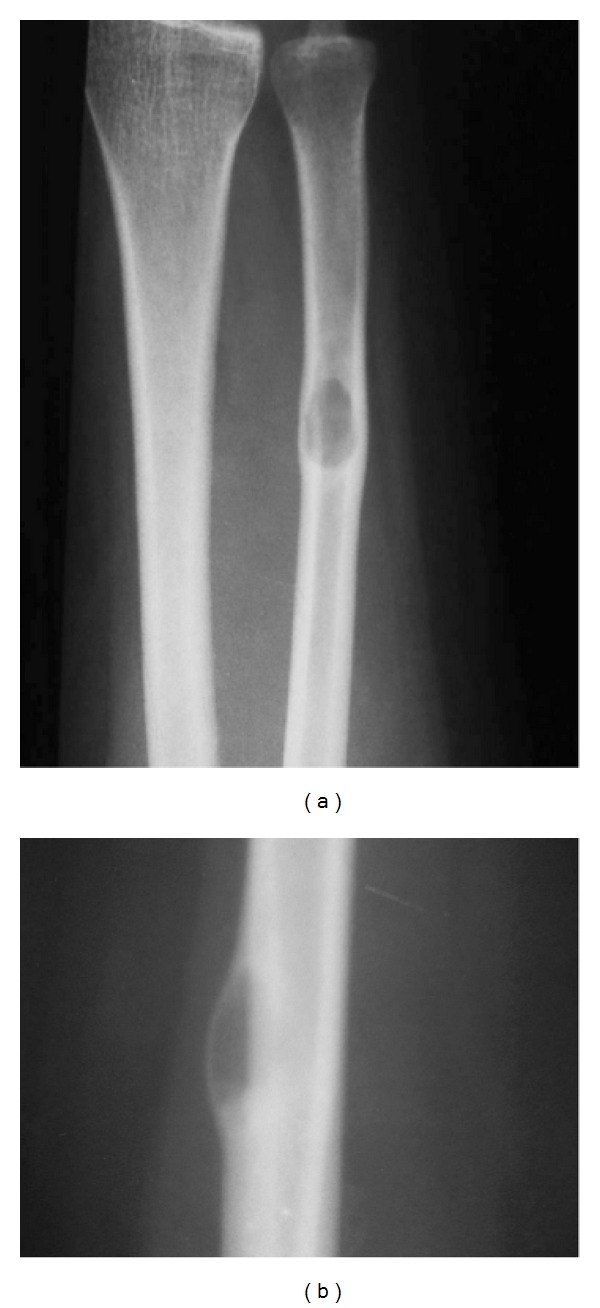
(a) antroposterior radiography revealed osteolytic well-defined lesion without marginal sclerosis and cortical destruction. (b) lateral radiography revealed eccentric exophytically osteolytic lesion without calcification or marginal sclerosis.

Antero-posterior and lateral (Figures 1(a) and 1(b)) X-rays revealed a well-defined eccentric osteolytic lesion in the distal diaphysis of left ulna with expansion of overlying cortex and without calcification. The overlying cortex was thinned with no cortical destruction. There was no marginal sclerosis.

**Figure 2:**
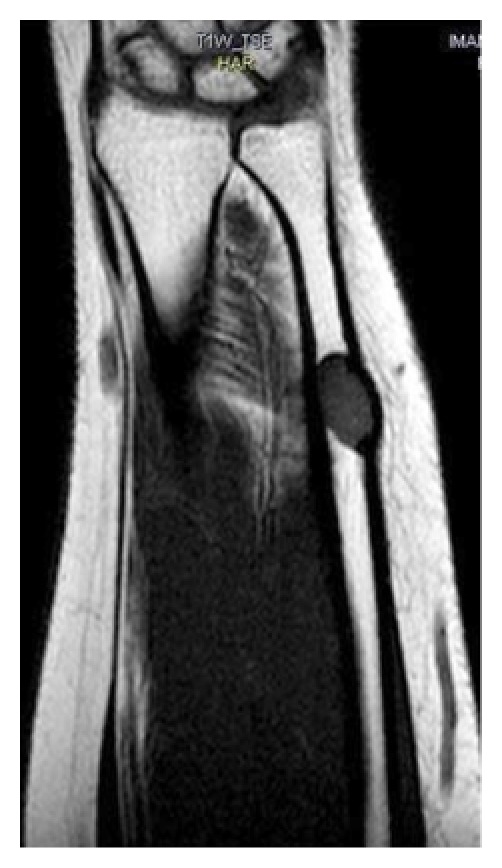
T1 W coronal image showed a well-defined low signal ovoid intramedullary lesion measured 2*1 cm in the distal diaphysis of left ulnar bone that was protruding exophytically from medial surface of ulnar bone.

**Figure 3:**
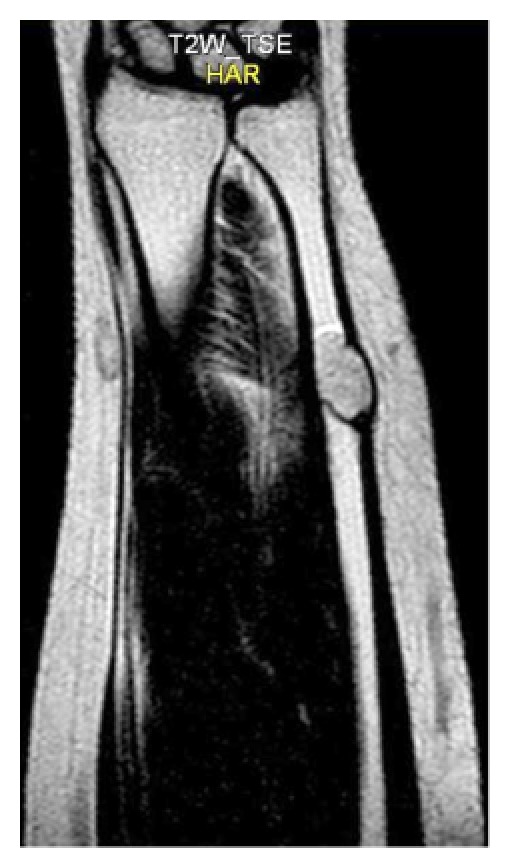
T2 W coronal image showed a well-defined high signal ovoid intramedullary lesion measured 2*1 cm in the distal diaphysis of left ulnar bone that was protruding exophytically from medial surface of ulna bone.

**Figure 4:**
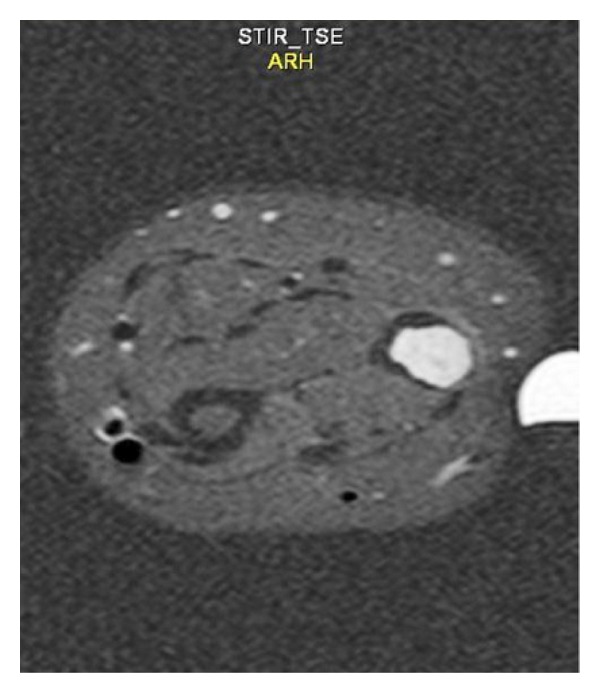
Fat-saturated fast spin-echo T2-weighted axial image showed a well-defined high signal ovoid intramedullary lesion measured 2*1 cm in the distal diaphysis of left ulnar bone that was protruding exophytically from medial surface of ulna bone.

Magnetic resonance imaging showed a well-defined ovoid intramedullary lesion measured 2 by 1 cm in the distal diaphysis of left ulnar bone that was exophytically protruding from the medial surface of left ulnar bone. The lesion was of low signal intensity on T1-weighted images (Figure 2) and high intensity signal on the T2-weighted (Figure 3) and fat-saturated fast spin-echo T2-weighted images (Figure 4).

**Figure 5:**
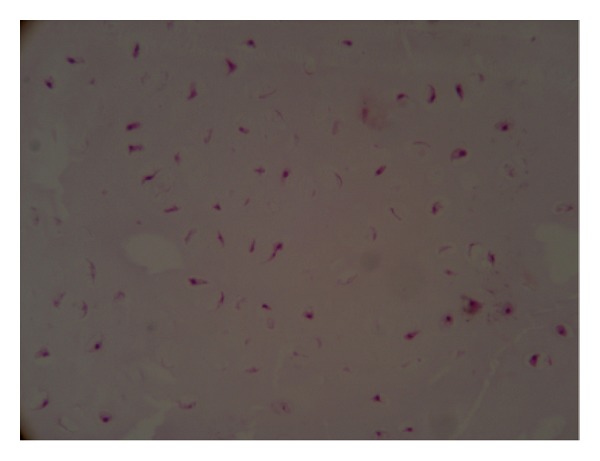
Histopathology showed a cartilaginous tissue with uniformly sized chondrocytes located in the round lacunae in a myxoid matrix.

Complete resection of the epiphytic cartilage cap with intramedullary curettage was performed. Histopathology showed a cartilaginous tissue with uniformly sized chondrocytes in a myxoid matrix located in the round lacunae, compatible with enchondroma and with no evidence of cytological dysplastic cell (Figure 5). The postoperative period was not eventful and after 5 months of followup there was no sign of recurrence.

## 3. Discussion

Enchondroma protuberans originates from the cartilage in the medullary cavity. It is defined as a protruded enchondroma that is beyond the cortex. There were 13 reported cases of the enchondroma protuberans in the literature and all were located in the humerus, ribs, and hand regions [1–10].

Enchondroma almost always appears as a well-defined osteolytic lesion that is usually located within the metadiaphysis of long bone. Due to bone expansion the cortex may appear thin, but usually remains intact. Enchondroma rarely expands through the cortex, and if accompanied by a cortical defect, will result in enchondroma protuberans [10].

Radiologically, enchondroma protuberans is presented as a well-defined intramedullary osteolytic lesion and may be accompanied by fine matricidal calcification, cortical expansion and cortical defect, and round well-defined soft-tissue expansion [1, 2, 6, 10].

The typical presentation of enchondroma protuberans in MR images is as a well-defined intramedullary lesion with low signal intensity in T1-W image and high signal intensity in T2-W and STIR sequences accompanied by cortical expansion and cortical defect [1, 2, 6, 10].

Although this benign tumor is usually diagnosed by conventional radiography, but according to X-ray findings in our and two previous case reports and also by An et al., sometimes it is not possible to be diagnosed based on conventional X-ray alone [10]. The conventional radiographs may not be able to detect protruding mass or cortical expansions. MR images can reveal the connection between the intramedullary portion and the exophytic protrusion better that conventional radiography. Also MR images can clearly delineate the cortical defect, which is essential in the diagnosis of enchondroma protuberans [1, 2, 6, 10]. Magnetic resonance imaging studies of our case report was similar to the previously reported cases of enchondroma protuberans [1, 2, 6, 10].

Enchondroma protuberans should be considered in the differential diagnosis of osteochondroma, Enchondroma, and periosteal chondroid tumors. Periosteal chondroma is benign cartilaginous tumor of periosteal origin, which occurs in young adults. Osteochondroma is continuous with the bone, which it originates and also contains a dense osteoid formation in the cortex and medulla.

The absence of cartilaginous cap and underlying trabecular bone differentiates it from osteochondroma and delineation of contiguous intramedullary involvement can be a strong indicator to differentiate it from periosteal chondroma. Because it has a potential risk of transformation to chondrosarcomatous, it is important to accurately diagnose and plane for a surgical treatment. To surgically treat the osteochondroma, excision of the cap is sufficient. But in patients with enchondroma protuberans, resection of the exophytic cartilage mass should be accompanied by intramedullary curettage [8].

## 4. Conclusion

Enchondroma protuberans should be considered in the differential diagnosis of osteochondroma, enchondroma, and periosteal chondroid tumors.
